# Esophagogastric Junction Outflow Obstruction Is Likely to Be a Local Manifestation of Other Primary Diseases: Analysis of Single-Center 4-Year Follow-Up Data

**DOI:** 10.3390/diagnostics13142329

**Published:** 2023-07-10

**Authors:** Yan Wang, Ting Yu, Feng Zhu, Ying Xu, Yun Bao, Ling Zhang, Lin Lin, Yurong Tang

**Affiliations:** 1 Department of Gastroenterology, The First Affiliated Hospital with Nanjing Medical University, Nanjing 210029, China; wangyan7733@njmu.edu.cn (Y.W.); njmuyt@163.com (T.Y.); zfxszy@sina.com (F.Z.); xycrystal0419@163.com (Y.X.); boring920316@outlook.com (Y.B.); zl201611102@163.com (L.Z.); lin9100@aliyun.com (L.L.); 2 Department of Gastroenterology and Hepatology, South China Hospital, Health Science Center, Shenzhen University, Shenzhen 440307, China; 3 Department of Gastroenterology, Southeast University Zhongda Hospital, Nanjing 210029, China

**Keywords:** esophagogastric junction outflow obstruction, achalasia cardia, high-resolution manometry, per-oral endoscopic myotomy, pneumatic dilation

## Abstract

Background: Whether esophagogastric junction outflow obstruction (EGJOO) is a variant of achalasia cardia (AC) or an esophageal motility state of certain organic or systemic diseases remains controversial. We aimed to investigate the differences between EGJOO and AC in clinical characteristics and outcomes through a 4-year follow-up. Methods: Patients diagnosed with primary EGJOO or AC were included. Based on the presence of concomitant disease, EGJOO patients were divided into a functional and an anatomical EGJOO group; similarly, patients with AC were divided into an AC with organic disease group and a true AC group. Disease characteristics and high-resolution manometry (HRM) parameters were retrospectively compared between the groups, and the development of organic diseases that could affect esophageal motility disorders and responses to treatment were examined during the follow-up. Symptom relief was defined as an Eckardt score of ≤3 after the treatment. Results: The study included 79 AC patients and 70 EGJOO patients. Compared with patients with AC, EGJOO patients were older, had shorter disease duration, a lower Eckardt score, and were more likely to have concurrent adenocarcinoma of the esophagogastric junction (AEG) and autoimmune disease (*p* < 0.05 for all). The severity of dysphagia and Eckardt scores were higher in the anatomical EGJOO group than in the functional EGJOO group. Significant differences were seen in HRM parameters (UES residual pressure, LES basal pressure, and LES residual pressure) between AC and EGJOO patients. However, no significant differences in HRM parameters were observed between the functional EGJOO and anatomical EGJOO groups. Sixty-seven (95.71%) patients with EGJOO and sixty-nine (87.34%) patients with AC experienced symptom relief (*p* = 0.071). Among patients achieving symptom relief, a relatively large proportion of patients with EGJOO had symptom relief after medications (37/67, 55.22%), the resolution of potential reasons (7/67, 10.45%), and spontaneous relief (15/67, 22.39%), while more patients with AC had symptom relief after POEM (66/69, 95.65%). Among EGJOO patients achieving symptom relief, more patients (7/20, 35%) with anatomical EGJOO had symptom relief after the resolution of potential reasons for EGJOO, while more patients (32/47, 68.09%) with functional EGJOO had symptom relief with medications. Conclusions: Concurrent AEG and autoimmune diseases are more likely in EGJOO than in AC. A considerable part of EGJOO may be the early manifestation of an organic disease. Anatomical EGJOO patients experience symptom improvement with the resolution of primary diseases, while most functional EGJOO patients experience symptom relief with pharmacotherapy alone or even without any treatment.

## 1. Introduction

The development of high-resolution manometry (HRM) has led to better identification of the various esophageal motility disorders, which are now clearly defined and classified in the Chicago Classification [1]. In our clinical work, we have often found secondary conditions in patients with esophageal motility disorders, leading us to question whether esophageal motility disorders could be a local manifestation of other primary diseases. In one previous study, more than 80% of patients with absent esophageal contractility had a systemic autoimmune rheumatic disease [2], leading the authors to suggest that esophageal dysmotility may be the first presentation of autoimmune diseases in some patients. Other studies have shown that peristaltic dysfunction is common in gastroesophageal reflux disease (GERD) [3] and can be improved with the relief of the esophageal reflux burden [4].

Esophageal hypermotility disorders include achalasia cardia (AC), esophagogastric junction outflow obstruction (EGJOO), jackhammer esophagus (JE), and distal esophageal spasm (DES) [1]. AC, which has been widely studied, is now considered to be caused by the impairment of esophageal inhibitory pathways, with no recognized organic basis [5]. JE and DES are uncommon esophageal motility disorders that have sometimes been found to be related to eosinophilic esophagitis, GERD, or vagus nerve stimulation [6,7]. EGJOO, which is only slightly less prevalent than AC according to the data at our center, often presents with similar features, that is, dysphagia, regurgitation, and retrosternal pain [8]. Some researchers believe that EGJOO is only an early stage of AC,10 but others consider EGJOO to be a distinct entity that needs to be treated differently from AC [9]. In our clinical practice, we have found that a considerable proportion of EGJOO patients have organic or systemic diseases. Endoscopic minimally invasive techniques such as pneumatic dilation (PD) and per-oral endoscopic myotomy (POEM), which have proven effective in the treatment of AC [5], are now also used for the treatment of EGJOO [10,11]. In general, however, there is still insufficient knowledge about EGJOO and only limited treatment experience.

The aim of this retrospective study was to review the symptoms, investigation results, treatment response, and follow-up data of patients with EGJOO without organic abnormalities diagnosed using HRM 4 years ago at our hospital and compare the differences between patients with EGJOO and AC. The findings of this study may help clinicians differentiate between these two similar esophageal motility disorders and thus help improve their management.

## 2. Materials and Methods

### 2.1. Study Design

The data of consecutive adult patients (age > 18 years) who underwent HRM for any indication between January 2017 and December 2018 at the Gastrointestinal Motility Center of our hospital were retrospectively reviewed. Patients whose HRM findings met Chicago Classification 3.0 criteria for EGJOO and AC were eligible for inclusion in this study. A telephone follow-up was conducted in December 2022. Patients with incomplete manometry studies, a history of upper gastrointestinal tract (including esophagogastric junction (EGJ)) surgery, or organic disease that may affect esophageal motility were excluded. Patients’ symptoms and treatments and the results of endoscopy, barium esophagogram, and computed tomography (CT) were retrieved from electronic medical records. Patients with EGJOO were classified as anatomical EGJOO if a potential anatomical explanation was identified during follow-up, including esophageal organic disease (hiatal hernia, esophageal ulcer, esophagitis Los Angeles grade C/D, esophageal fistula); concomitant autoimmune disease; herpes zoster; or adenocarcinoma of the esophagogastric junction (AEG). Similarly, based on potential anatomical explanations identified during follow-up, patients with AC were classified as AC with organic disease or true AC. The Eckardt score was calculated using the main symptoms (dysphagia, regurgitation, chest pain, and weight loss) [12]. Symptom relief was defined as an Eckardt score of ≤3 after treatment.

The study protocol was approved by the Ethical Committee of our hospital (No. 2021-SR-197).

### 2.2. High-Resolution Manometry

Patients were instructed to stop proton pump inhibitors (PPIs) and prokinetic drugs for at least 2 weeks and fast for at least 6 h before HRM. The HRM catheter (Given Imaging) was positioned by an experienced nurse and then inserted into the esophagus. The HRM protocol comprised baseline pressure recording for 1 min followed by pressure recordings during 10 single swallows of 5 mL warm water each [13]. At the time of the HRM study, dry swallows, double swallows, and swallows that occurred within 20 s of a prior swallow were all disregarded. The pressure topography was obtained using the manometry system and analyzed according to the Chicago Classification, version 3.0 (ManoView software). The pressure and relaxations of the lower esophageal sphincter (LES) were located, measured, and analyzed. Based on pressure differentials between intraesophageal and intragastric pressure marks, the proximal and distal borders were marked. The HRM metrics analyzed were upper esophageal sphincter (UES) pressure, LES pressure, and integrated relaxation pressure (IRP). AC was diagnosed as an IRP > 15 mmHg and 100% failed peristalsis or if spasms were found, and EGJOO was diagnosed when an IRP > 15 mmHg and type I–III ACs were not found.

### 2.3. Statistical Analysis

Continuous data were expressed as the means ± standard deviations or medians (with interquartile range) and compared using the unpaired Student’s *t*-test or the Mann–Whitney *U* test, as appropriate. Categorical variables were expressed as frequencies and proportions and compared using the chi-square test or the Fisher exact test. Statistical significance was at *p* < 0.05.

## 3. Results

### 3.1. Baseline Characteristics of Patients with AC vs. EGJOO

**Figure 1:**
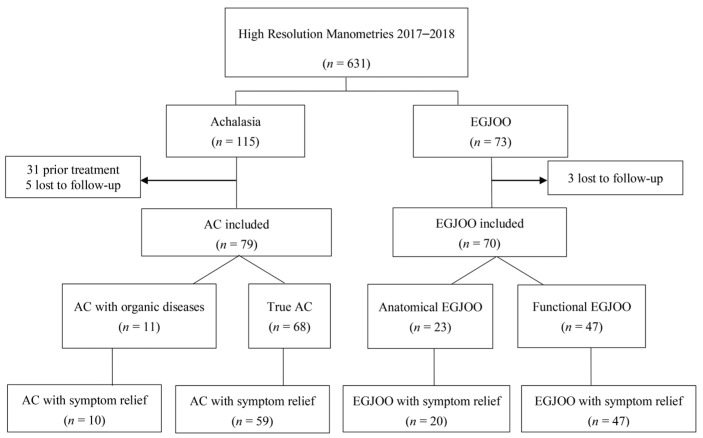
Enrollment of study patients. EGJOO, esophagogastric junction outflow obstruction.

**Figure 2:**
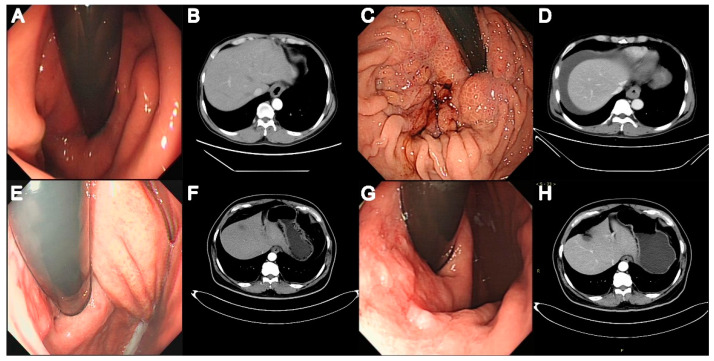
Endoscopic and computed tomography (CT) scan images of two patients with EGJOO and subsequent adenocarcinoma of the esophagogastric junction (AEG). Patient 1 was diagnosed with cardia inflammation using endoscopy (**A**) and pathology. Thickened cardiac wall and slight stricture of the esophagus are seen on CT (**B**) performed at the initial visit. Six months later, AEG (T3N0M1) was diagnosed. Endoscopy shows coarse, hyperemic, and easily bleeding cardia mucosa (**C**). CT shows an obviously thickened cardiac wall (**D**). In Patient 2, endoscopy performed at the initial visit (**E**) shows cardia mucosa hyperemia; CT (**F**) shows a slightly thickened cardiac wall. Endoscopy (**G**) performed one-and-a-half years later shows high-grade localized intraepithelial neoplasia in the gastric mucosa; this was confirmed on pathology. CT shows thickened and enhanced cardiac wall (**H**).

A total of 149 patients (79 with AC, 70 with EGJOO) were included in this study. Figure 1 shows the patient selection process. The median follow-up was for 28 (22–36) months. Of the 79 patients with AC, 5 (6.33%) had AC type 1, and 74 (93.67%) had AC type 2. Table 1 lists the conditions concomitantly present in patients with EGJOO and AC. Concomitant AEG and autoimmune diseases were significantly more common in EGJOO (*p* = 0.021 and *p* = 0.026). The concomitant autoimmune diseases in EGJOO included rheumatoid arthritis (three patients), Sjogren syndrome (two patients), and dermatomyositis (two patients). One patient with AC had concomitant hypothyroidism. A family history of cancer of the esophagus, EGJ, or stomach was significantly more common in patients with EGJOO (*p* = 0.002). Of note, 5/70 (7.14%) patients with EGJOO were diagnosed with AEG during follow-up. Figure 2 shows the endoscopic and CT images of the two patients with EGJOO and subsequent AEG.

Of the 70 patients with EGJOO, 23 were classified as anatomical EGJOO and 47 as functional EGJOO. Of the 79 patients with AC, 11 were classified as AC with organic disease and the other 68 as true AC. At baseline, patients with EGJOO were older and had shorter disease courses than patients with AC (both *p* < 0.001; Table 2). Baseline dysphagia, regurgitation, and weight loss were less severe in patients with EGJOO than in patients with AC, and therefore, the baseline Eckardt score was lower in the former, especially among patients with functional EGJOO. The baseline dysphagia score and the total Eckardt score were higher in the anatomical EGJOO group than in the functional EGJOO group. Baseline odynophagia and globus were more common in EGJOO patients, while nausea and vomiting were more common in AC patients. No statistically significant differences were found in clinical characteristics and symptoms between the AC with organic disease group and the true AC group.

### 3.2. Baseline HRM Parameters in Patients with AC vs. EGJOO

Table 3 compares the manometric parameters in patients with EGJOO and AC. LES basal pressure and IRP were significantly lower and the rate of LES relaxation was significantly higher in the EGJOO group than in the AC group. UES parameters also showed significant differences between the two groups, with UES mean basal pressure, UES residual pressure, and recovery time being significantly lower in the EGJOO group than in the AC group. Manometric variables that did not differ significantly between the groups included the UES relaxation time of nadir and hiatus hernias. No statistically significant differences in HRM parameters were observed between the functional EGJOO group and the anatomical EGJOO group.

### 3.3. Treatments and Outcomes in EGJOO vs. AC

**Figure 3:**
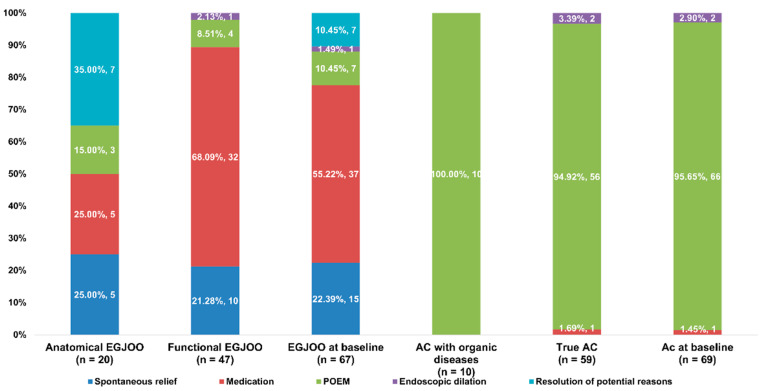
Proportion of patients achieving symptom relief with different therapies in the EGJOO and AC groups. EGJOO, esophagogastric junction outflow obstruction; AC, achalasia cardia; POEM, per-oral endoscopic myotomy.

Symptom relief was achieved in 20/23 (86.96%) patients with anatomical EGJOO, 47/47 (100%) patients with functional EGJOO, 10/11 (90.91%) patients with AC with organic diseases, and 59/68 (86.76%) patients with true AC. Figure 3 summarizes the treatments used. In the anatomical EGJOO group, symptom relief was achieved with a resolution of potential reasons in 7/20 (35%) patients (in three patients after the resolution of autoimmune disease, in three patients after the resolution of AEG, and in one patient after the resolution of herpes zoster). The remaining patients in the anatomical EGJOO group had symptom relief after various other management approaches: 5/20 (25%) patients had symptom relief after medications (PPIs, smooth muscle relaxants, calcium channel blockers, and prokinetics), 5/20 (25%) patients had symptom relief without any treatment (i.e., spontaneous relief), and 3/20 (15%) patients had symptom relief after POEM. Two anatomical EGJOO patients with persistent symptoms had AEG and received laparoscopic subtotal gastrectomy, while one patient with persistent symptoms refused any treatment. In the functional EGJOO group, 32/47 (68.09%) patients had symptom relief with medications, 10/47 (21.28%) patients achieved spontaneous symptom relief, 4/47 (8.51%) patients had symptom relief after POEM, and 1/47 (2.13%) patients had symptom relief after PD. In the AC group, symptom relief was achieved with POEM in 66/69 (95.65%) patients, with PD in 2/69 (2.90%) patients, and with medications (PPI and prokinetics) in 1/69 (1.45%) patients. The remaining 10/79 (12.66%) patients in the AC group underwent POEM but had no relief of symptoms. Statistically significant differences were seen between the AC and EGJOO groups in the proportions of patients experiencing spontaneous symptom relief, relief with medications, relief with the resolution of potential reasons, and relief with POEM (all *p* < 0.001). The differences between the anatomical EGJOO and functional EGJOO groups in the proportions of patients experiencing symptom relief via medications and via the resolution of potential reasons were statistically significant (both *p* < 0.001). No significant differences in the rates of relief were observed between the AC with organic diseases group and the true AC group.

## 4. Discussion

In this study, we analyzed the baseline and 4-year follow-up data of 149 patients with primary diagnoses of EGJOO or AC. There were only minor differences in symptoms between the two groups, but outcomes and responses to treatment were obviously distinct.

We found that the main symptoms, assessed using the Eckardt score, were milder in patients with EGJOO than in patients with AC. Similar to prior studies, dysphagia and regurgitation were the main complaints of EGJOO patients in our study [14,15]. Vomiting was less common in EGJOO than in AC, suggesting that the severity of EGJ obstruction is lower in EGJOO. Odynophagia and globus were more common in EGJOO than in AC, indicating that the symptoms of EGJOO are heterogeneous.

The causes of EGJOO remain unclear, but a considerable proportion may be secondary to other conditions. In a previous study, among patients with secondary esophageal motility disorders, 7/16 (43.75%) had EGJOO [16], suggesting that EGJOO with an organic origin is not uncommon. In our study, concomitant autoimmune diseases were more common in the EGJOO group than in the AC group. The main strengths of this study are the large sample size compared with previous studies and the consideration of a greater number of potential reasons for anatomical EGJOO. Further clinical investigations are required to clarify the pathological mechanisms of EGJOO.

AC may be associated with the degeneration of the myenteric plexus or with a combination of infectious, autoimmune, and familial factors; this is distinct from the pathophysiology of EGJOO [17]. In the present study, patients with EGJOO were more likely to have concomitant AEG; autoimmune disease; and a family history of esophageal, EGJ, or gastric cancer. Recent clinical guidelines recommend some form of surveillance for cancer in patients with AC [5]; similar surveillance may also be justified in patients with EGJOO.

The overall clinical characteristics and HRM parameters were similar in anatomical EGJOO and functional EGJOO patients, which indicates that symptomatology and HRM metrics may not be sufficient to determine whether an organic disease exists. Considering the relatively large proportion of EGJOO patients who have concomitant diseases, detailed investigations are warranted in EGJOO patients. Our results suggest that tumors, autoimmune diseases, and abnormal esophageal anatomy are common in anatomical EGJOO; hence, HRM could provide more evidence for pursuing computed tomography, autoimmune status examination, and upper gastrointestinal endoscopy in patients identified as having EGJOO.

It is not surprising that the HRM metrics of LES basal pressure and IRP were lower in EGJOO patients. It was reported that elevated LES pressure is associated with more severe dysphagia in patients with hypercontractile peristalsis [18]. In this study, UES residual pressure and UES recovery time were also lower in EGJOO than in AC. Pierre and colleagues showed that UES residual pressure increased progressively from asymptomatic controls to EGJOO and to AC [19]. Furthermore, nadir UES residual pressure can stratify EGJOO into motor and mechanical subgroups, which might help predict treatment responses [20]. The underlying mechanism may be that patients with impaired LES relaxation are more likely to have a UES abnormality than patients with normal LES relaxation [21]. The differences in HRM metrics between patients with EGJOO and AC indicate that EGJOO might not be a variant of AC and is actually a distinct entity that involves abnormity in both the UES and LES. Recent studies have shown that provocative tests such as the rapid drink challenge and multiple rapid swallows may yield additional information (such as intact peristalsis during the test) that would help identify EGJOO [22]. Functional luminal imaging probes might also help stratify EGJOO patients for appropriate management [23].

Various treatments have been used for EGJOO, including medications, endoscopic dilation, botulinum toxin injections, and POEM. In our study, a fairly large proportion (35%) of patients with anatomical EGJOO responded to the treatment of potential reasons, and the majority of patients with functional EGJOO (68.09%) improved with medications. Thus, the treatment of the underlying primary disease might improve symptoms in anatomical EGJOO patients, while functional EGJOO may benefit from medications and close follow-ups. The proportion of EGJOO patients achieving spontaneous relief (22.39%) was relatively low compared with earlier studies [24,25], probably because only symptomatic EGJOO patients were included in our study.

POEM has been recognized as an efficacious therapy of the LES [5]. Among AC patients in this study, the treatment success rate for POEM was 86.84% (66/76); this rate is consistent with rates of 80–97% reported by other authors [26]. Of the EGJOO patients with a favorable outcome, seven (10.45%) underwent POEM. Tissue biopsies show that the neuroimmunological profile of EGJOO is completely different from that of AC [9], which could explain the discrepancy in treatment outcomes between these two groups. Considering the damage to the anti-reflux barrier and the high prevalence of reflux esophagitis in patients post-POEM [26], clinicians need to be cautious in treating EGJOO with POEM.

To the best of our knowledge, there is no previous published research comparing the clinical characteristics, treatments, and outcomes of anatomical EGJOO versus functional EGJOO or EGJOO versus AC in Chinese populations, where 24.2% of patients with esophageal dysphagia but normal endoscopy show evidence of EGJOO [27]. A better understanding of the differences between EGJOO and AC will help in the selection of specific and efficient therapies.

Several limitations exist in this study. First, post-treatment follow-up data were not obtained for many patients with EGJOO. Because of the high rate of symptom relief, most EGJOO patients were not willing to undergo HRM or endoscopy again. Secondly, a logistic regression analysis to identify predictors of favorable treatment responses in patients with EGJOO was not performed because of the small number of patients with unfavorable responses.

**Figure 4:**
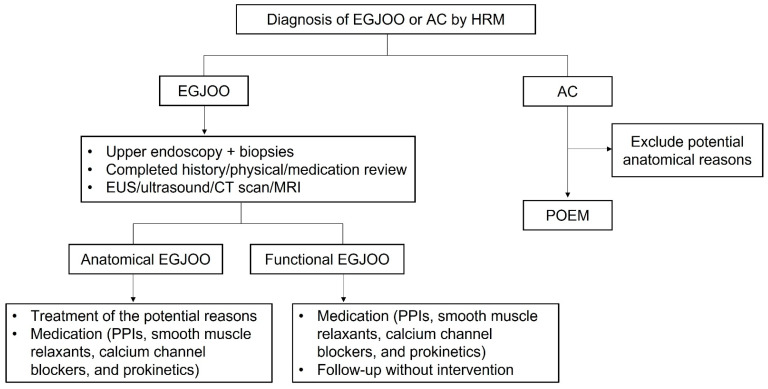
Diagnosis and treatment algorithm for patients with EGJOO and AC. EGJOO, esophagogastric junction outflow obstruction; AC, achalasia cardia; HRM, high-resolution manometry; EUS, endoscopy ultrasound; CT, computed tomography; MRI, magnetic resonance imaging; PPIs, proton pump inhibitors; POEM, per-oral endoscopic myotomy.

To conclude, the main symptoms are milder in EGJOO than in AC. EGJOO patients are more likely to have coexisting AEG and autoimmune diseases. Most EGJOO patients have symptom relief with the resolution of primary diseases or medications alone, or even without any specific treatment. For patients with EGJOO, clinicians should take concomitant conditions into consideration. Even if various examinations such as CT and immune indicators are normal at the initial diagnosis, a close follow-up is required. Moreover, invasive treatments for EGJOO should be avoided as much as possible. Based on our results, we can summarize a diagnosis and treatment algorithm for EGJOO and AC (Figure 4). Patients with anatomic EGJOO should first be considered for the treatment of potential reasons, either with medication or follow-up without intervention. Patients with functional EGJOO can be treated with medication, and about 20% of patients experience spontaneous relief. The POEM treatment is the first-line treatment for patients with AC.

## Figures and Tables

**Table 1:** Concomitant conditions detected in patients with EGJOO and AC.

	EGJOO (*n* = 70)	AC (*n* = 79)	*p*
Adenocarcinoma of the esophagogastric junction	5 (2.86%)	0 (0%)	0.021 *
Autoimmune disease	7 (10%)	1 (1.27%)	0.026 *
Esophageal organic disease †	5 (2.86%)	5 (6.33%)	0.999
Herpes zoster	6 (8.57%)	5 (6.33%)	0.601
Family history ‡	13 (18.57%)	2 (2.53%)	0.002 *

EGJOO, esophagogastric junction outlet obstruction; AC, achalasia cardia. Note: Data are expressed as *n* (%). † Esophageal ulcer, esophagitis Los Angeles grade C/D, or esophageal fistula. ‡ Cancer of the esophagus, esophagogastric junction, or stomach. * *p* < 0.05.

**Table 2:** Comparison of demographics, clinical characteristics, and symptoms between AC and EGJOO patients.

	Anatomical EGJOO (*n* = 23)	Functional EGJOO (*n* = 47)	EGJOO at Baseline (*n* = 70)	AC with Organic Diseases (*n* = 11)	True AC (*n* = 68)	AC at Baseline (*n* = 79)
Age (year)	56.57 ± 15.88	52.49 ± 13.81	53.83 ± 14.53	46.27 ± 15.12	44.47 ± 15.85	44.72 ± 15.67 ^c^
Male	12 (52.17%)	19 (40.43%)	31 (44.29%)	5 (45.45%)	35 (51.47%)	40 (50.63%)
BMI (kg/m^2^)	22.84 ± 4.58	22.32 ± 3.30	22.49 ± 3.76	20.81 ± 3.55	22.04 ± 5.24	21.86 ± 5.04
Duration (months)	24.0 (12.0–54.0)	24.0 (12.0–36.0)	24.0 (12.0–36.0)	96.0 (60.0–336.0)	48.0 (18.0–120.0) ^b^	60.0 (24.0–120.0) ^c^
Eckardt score						
Dysphagia	1.50 ± 1.26	0.67 ± 0.90 ^a^	0.95 ± 1.10	2.55 ± 0.69	2.41 ± 0.80	2.43 ± 0.78 ^c^
Regurgitation	0.68 ± 0.72	0.61 ± 0.77	0.63 ± 0.75	0.91 ± 0.94	0.97 ± 0.90	0.96 ± 0.90 ^c^
Chest pain	0.45 ± 0.67	0.55 ± 0.77	0.52 ± 0.73	0.91 ± 1.04	0.63 ± 0.91	0.67 ± 0.93
Weight loss	0.23 ± 0.53	0.24 ± 0.43	0.23 ± 0.46	0.64 ± 0.81	0.57 ± 0.87	0.58 ± 0.86 ^c^
Total	2.86 ± 1.64	2.05 ± 1.17 ^a^	2.33 ± 1.39	5.00 ± 1.73	4.59 ± 1.74	4.65 ± 1.73 ^c^
Heartburn	6 (26.09%)	14 (29.79%)	20 (28.57%)	5 (45.45%)	16 (23.53%)	21 (24.71%)
Nausea	5 (21.74%)	5 (10.64%)	10 (14.28%)	5 (45.45%)	26 (38.24%)	31 (39.24%) ^c^
Vomiting	6 (26.09%)	4 (8.51%)	10 (14.28%)	7 (63.64%)	32 (47.06%)	39 (49.37%) ^c^
Epigastric pain	6 (26.09%)	8 (17.02%)	14 (20%)	3 (27.27%)	12 (17.65%)	15 (18.99%)
Odynophagia	5 (21.74%)	12 (25.53%)	17 (24.29%)	0 (0%)	2 (2.94%)	2 (2.53%) ^c^
Cough	3 (13.04%)	5 (10.64%)	8 (11.43%)	2 (18.18%)	4 (5.88%)	6 (7.59%)
Aspiration	0 (0%)	0 (0%)	0 (0%)	2 (18.18%)	2 (2.94%)	4 (5.06%)
Hoarseness	1 (4.35%)	3 (6.38%)	4 (5.71%)	0 (0%)	0 (0%)	0 (0%) ^c^
Belching	10 (43.48%)	16 (34.04%)	26 (37.14%)	4 (36.36%)	18 (26.47%)	22 (27.85%)
Globus	3 (13.04%)	8 (17.02%)	11 (15.71%)	1 (9.09%)	1 (1.47%)	2 (2.53%) ^c^

AC, achalasia cardia; EGJOO, esophagogastric junction outlet obstruction; BMI, body mass index. Note: Data are expressed as mean ± SD or medians (interquartile range) or number (*n*) and percentage (%). ^a^ *p* < 0.05 between anatomical and functional EGJOO. ^b^ *p* < 0.05 between AC with organic diseases and true AC. ^c^ *p* < 0.05 between EGJOO and AC at baseline.

**Table 3:** High-resolution manometry parameters in patients with AC and EGJOO.

	Anatomical EGJOO (*n* = 23)	Functional EGJOO (*n* = 47)	EGJOO at Baseline (*n* = 70)	AC with Organic Diseases (*n* = 11)	True AC (*n* = 68)	AC at Baseline (*n* = 79)
UES mean basal pressure	47.5 (30.6–58.3)	58.1 (43.0–76.2)	50.0 (38.2–70.8)	50.3 (41.3–67.3)	65.6 (41.0–87.4)	62.3 (41.2–86.7)
UES residual pressure	1.5 (−1.9–7.4)	3.1 (−1.6–8.4)	2.1 (−1.7–7.7)	10.8 (3.0–14.8)	9.4 (5.7–15.4)	9.4 (5.6–15.1) ^b^
UES relaxation time to nadir	225.0 (129.0–280.0)	150.0 (101.0–220.0)	164.0 (110.3–234.5)	297.0 (201.0–491.0)	139.5 (101.8–209.0) ^a^	161.0 (102.0–252.5)
UES recovery time	390.0 (331.0–592.0)	519.0 (363.0–619.0)	464.0 (344.5–608.0)	457.0 (379.0–594.0)	556.0 (416.0–699.5)	537.0 (402.5–682.0) ^b^
LES basal pressure (proximal)	42.87 ± 2.83	41.98 ± 3.08	42.27 ± 3.01	44.18 ± 3.28	44.82 ± 3.34	44.74 ± 3.32 ^b^
LES basal pressure (distal)	46.57 ± 2.74	45.93 ± 2.83	46.14 ± 2.80	48.43 ± 2.68	48.05 ± 3.23	48.10 ± 3.15 ^b^
LES basal pressure (minimum)	22.37 ± 9.27	21.36 ± 8.82	21.69 ± 8.91	25.41 ± 12.07	29.95 ± 14.62	29.39 ± 14.34 ^b^
LES basal pressure (mean)	31.0 (25.1–37.2)	28.2 (24.9–37.4)	29.0 (25.1–37.3)	32.1 (23.9–44.5)	36.7 (28.3–48.3)	36.0 (28.2–48.2) ^b^
IRP	19.2 (16.5–21.8)	17.8 (16.5–21.6)	17.9 (16.5–21.7)	29.9 (21.6–38.5)	31.4 (22.0–37.6)	30.4 (22.1–37.6) ^b^
LES residual pressure (maximum)	25.5 (20.6–30.7)	23.2 (21.1–27.2)	23.7 (21.1–30.1)	39.8 (26.4–49.7)	41.0 (28.1–51.5)	40.6 (27.8–49.7) ^b^
Esophageal length	25.38 ± 2.17	25.14 ± 1.75	25.22 ± 1.89	26.51 ± 2.37	27.34 ± 2.63	27.24 ± 2.60 ^b^
LES length	3.69 ± 0.81	3.95 ± 1.05	3.87 ± 0.98	4.26 ± 1.29	3.45 ± 0.79	3.55 ± 0.90 ^b^
LES abdomen length	2.7 (2.6–4.1)	3.0 (2.4–3.6)	3.0 (2.4–3.6)	3.5 (2.5–4.8)	2.7 (2.1–3.3)	2.7 (2.1–3.4)
LES relaxation (%)	44.35 ± 11.50	43.45 ± 11.21	43.74 ± 11.23	7.56 ± 10.93	19.67 ± 17.44 ^a^	18.18 ± 17.19 ^b^
Hiatus hernia, *n* (%)	1 (4.35%)	0 (0%)	1 (1.43%)	0 (0%)	2 (2.94%)	2 (2.53%)

Note: Data are expressed as mean ± SD or medians (interquartile range). EGJOO, esophagogastric junction outlet obstruction; AC, achalasia cardia; UES, upper esophageal sphincter; LES, lower esophageal sphincter; IRP, integrated relaxation pressure. ^a^ *p* < 0.05 between AC with organic diseases and true AC. ^b^ *p* < 0.05 between EGJOO and AC at baseline.

## Data Availability

Not applicable.
